# Heavy metal and nutrient uptake in plants colonizing post-flotation copper tailings

**DOI:** 10.1007/s11356-017-0451-y

**Published:** 2017-10-23

**Authors:** Dorota Kasowska, Krzysztof Gediga, Zofia Spiak

**Affiliations:** 1 Department of Botany and Plant Ecology, Wrocław University of Environmental and Life Sciences, Grunwaldzki Square 24A, 50-363 Wrocław, Poland; 2 Department of Plant Nutrition, Wrocław University of Environmental and Life Sciences, Grunwaldzka Street 53, 50-357 Wrocław, Poland

**Keywords:** Tailings, Copper mining, Metal pollution, Element accumulation, Phytoremediation

## Abstract

Copper ore mining and processing release hazardous post-flotation wastes that are difficult for remediation. The studied tailings were extremely rich in Cu (1800 mg kg^−1^) and contaminated with Co and Mn, and contained very little available forms of P, Fe, and Zn. The plants growing in tailings were distinctly enriched in Cu, Cd, Co, Ni, and Pb, and the concentration of copper achived the critical toxicity level in shoots of *Cerastium arvense* and *Polygonum aviculare*. The redundancy analysis demonstrated significant relationship between the concentration of available forms of studied elements in substrate and the chemical composition of plant shoots. Results of the principal component analysis enabled to distinguish groups of plants which significantly differed in the pattern of element accumulation. The grass species *Agrostis stolonifera* and *Calamagrostis epigejos* growing in the tailings accumulated significantly lower amounts of Cu, but they also had the lowest levels of P, Fe, and Zn in comparison to dicotyledonous. *A. stolonifera* occurred to be the most suitable species for phytostabilization of the tailings with regard to its low shoot Cu content and more efficient acquisition of limiting nutrients in relation to *C. epigejos*. The amendments improving texture, phosphorus fertilization, and the introduction of native leguminous species were recommended for application in the phytoremediation process of the tailings.

## Introduction

Mining and processing of copper ores extensively alter the environment, and the main difficulty is a huge amount of flotation tailings which comprise even 96% of the mass of run-of-mine ores with worldwide production estimated to be two billion tons in 2011 (Gordon 2002; Onuaguluchi and Eren 2012). These tailings pose an enormous problem in EU countries, particularly in Poland where the copper production accounted over 421,000 t, which was accompanied by tailing deposition which amounted to 8.5 million tons in 2014 (Brown et al. 2016). The tailings consist mainly of very fine grinding rocks, and, as a result of the beneficiation process, they contain high concentrations of copper and other trace elements (Łuszczkiewicz 2000; BREF 2009; Wang et al. 2014). These kinds of wastes are usually deposited without any treatment in extensive ponds which pose opened sources of pollutants for neighboring ecosystems. The migration of heavy metals leads to the contamination of air, water, soil, and sediments, which negatively affects all organisms, including humans (Baycu et al. 2015; Venkateswarlu et al. 2016).

Among the technologies that may be employed in remediation of metal-contaminated lands and especially in the case of large sites, phytoremediation constitutes the most useful and cost-effective way that can be applied with minimum environmental impact (Khan et al. 2004). In the phytoextraction technique, certain plants are used (e.g., hyperaccumulators of trace elements) that have the extraordinary ability to take up and accumulate contaminants in their aboveground parts. “Phytomining,” however, has certain limitations and induces ecological risk because of introducing potentially toxic metals into the food chain or the improper disposal of contaminated biomass (Mench et al. 2010; Venkateswarlu et al. 2016). Reduction of metal mobility and bioavailability can be achieved due to the phytostabilization technique which uses suitable plant species and associated microbes for revegetation of contaminated sites. This enables the stabilization of the site area and pollution control, visual improvement, and removal of threats for herbivores and human beings (Wong 2003; Sheoran et al. 2010).

Establishing vegetation on abandoned metalliferous wastes is usually difficult because these artificial habitats create unfavorable conditions for plant growth. They are characterized by poor physical properties of substrate with unsuitable air-water conditions, high content of toxic metals, salinity, low fertility, and microbial activity (Forsberg 2008; Neuschütz 2009; Sheoran et al. 2010; Rybak et al. 2014). As a result, these wastelands are often devoid of any vegetation or have only sparse plant cover and are unable to create sustaining and healthy ecosystems. Especially, soils with high or contrasting metal contents impose strong selective pressure on colonizing plants which can persist in such habitats due to a wide range of adaptations (Baker 1981; Kinzel and Lechner 1992; Broadley et al. 2003). The lack of nutrients or their low availability is a similarly important selective factor, which hardly constrains vegetation development (Kazakou et al. 2008; Sheoran et al. 2010; Turnau et al. 2010). However, the problem of metal dispersion and nutrient deficiency can be solved by using clean topsoil to cover the waste substrate, but usually it must be transported from distant areas, which is costly, especially in the case of large wastelands. In this connection, revegetation of metalliferous wastes should be carried out with attentively selected plants that must be metal resistant and adapted to nutrient-deficient soils and can improve soil biological activity, grow quickly, and form dense canopies and root systems. These plants should be also adapted to local environmental conditions and be of native origin (Mench et al. 2010; Sheoran et al. 2010), so species spontaneously colonizing particular sites should be primarily considered and implemented for phytoremediation practices. Our main aims in the present study were (1) to evaluate heavy metal and nutrient contents in shoots of plants that colonize post-flotation copper tailings and juxtapose these data with chemical composition of the examined species growing on an unpolluted site, (2) to reveal the interactions between plant chemical composition and the specific properties of the copper tailings, and (3) to select plants and formulate general recommendations for the phytoremediation of these wastes. The tested hypothesis was whether the plant species that grow spontaneously on the copper tailings may be used as suitable organisms for their phytoremediation.

## Materials and methods

### Study sites

**Fig. 1:**
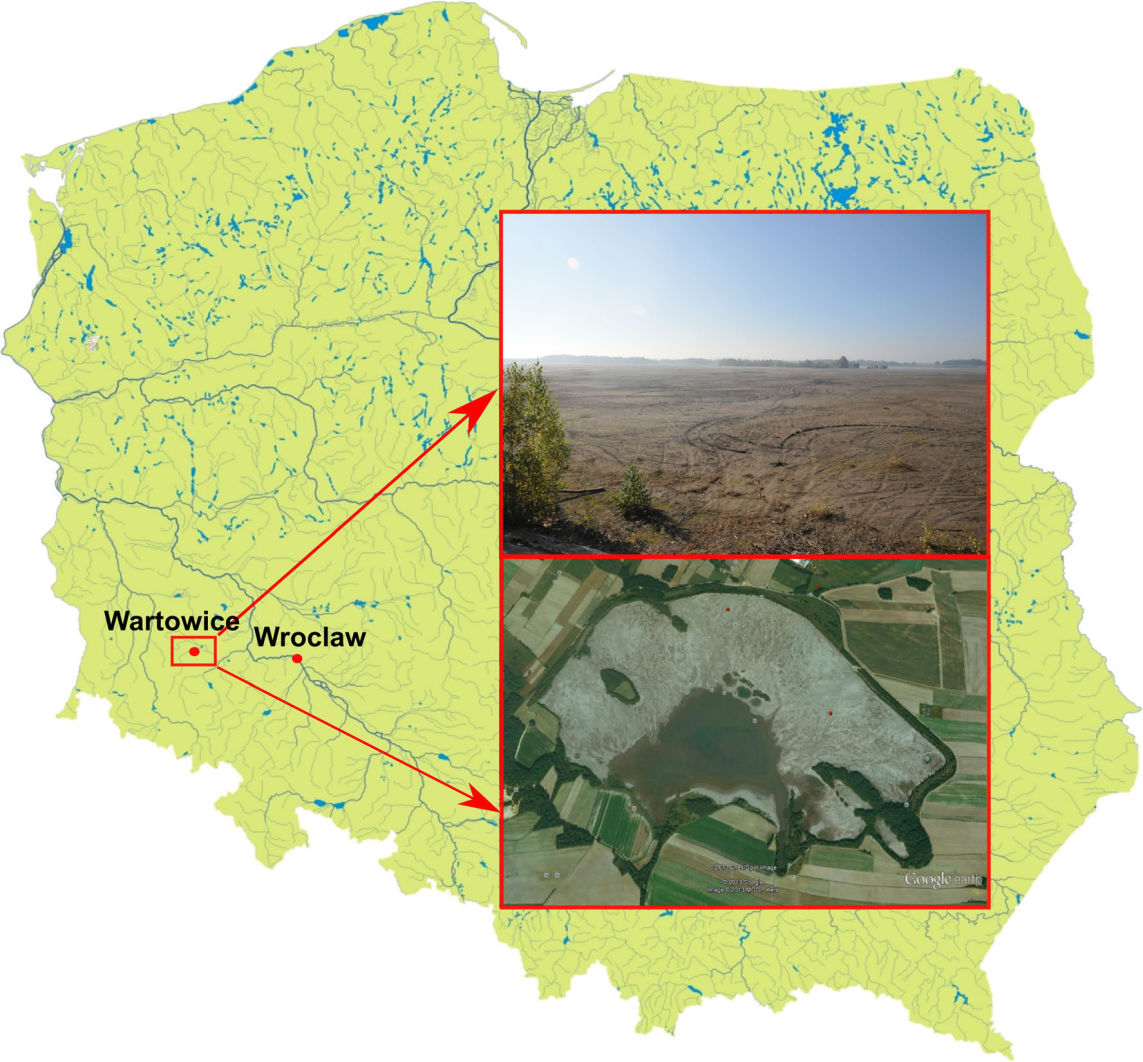
Location of the study sites and the view of “Wartowice 3” tailings pond with extremely poor vegetation

The studies were carried out in a “Wartowice 3” tailings pond area (51° 12′ 38.34″ N 15° 40′ 53.58″ E) which is located in Warta Bolesławiecka district, Lower Silesia province, SW Poland (Fig. 1). This pond covers about 232 ha and had been in mining activity for storing tailings from the copper ore flotation process until 1989. The main materials forming the tailings are silica (quartz), carbonate minerals (dolomite, calcite), and clay minerals, as well as to a lesser extent shale copper ores, marl, shale, and anhydrite.

Despite of natural succession lasting over 20 years, the pond’s surface remains almost bare with very poor vegetation. There are only infrequent and widely scattered stands, of a surface area of approx. 2 m^2^, of stunted specimens of *Pinus sylvestris* L. and *Populus tremula* L. as well as a few herbaceous species with dominance of *Agrostis stolonifera* L. Only the places that are overburden and with a little share of the copper tailings have greater and more diverse plant cover. A few attempts at the reclamation of the tailings were made in the past, but they were costly and ineffective, resulting in very sparse plant cover.

In comparison to the tailings pond, an urban wasteland with grassland and ruderal vegetation was chosen as a reference site which inhabited these plant species that lived in the pond. It was located in a relatively clean area nearby the Ślęza River in Wrocław city, SW Poland (Fig. 1).

### Sampling and chemical analysis

Because of the extremely poor vegetation that covers the pond, only five herbaceous plant species could be examined in this study, such as *Agrostis stolonifera* L. and *Calamagrostis epigejos* (L.) Roth (both from the Poaceae family), *Cerastium arvense* L. (Caryophyllaceae), *Polygonum aviculare* L. (Polygonaceae), and *Tussilago farfara* L. (Asteraceae). Samples of these species and substrates were collected from the tailings pond and the reference site, and they consisted of aboveground plant parts and copper tailings or reference soil taken from the plant rooting zone (10–20 cm depth). In the tailings pond, each species with substrate was collected randomly from three to five sampling sites. There were ten sampling sites in total with different compositions of collected species. At the reference site, the samples were taken randomly from four quadrates of a surface area 25 m^2^ with similar plant composition.

An analysis of granulometric composition of the copper tailings was carried out with use of the aerometric method. Organic carbon was determined by a CHNS analyzer from CE Instruments (Skjemstad and Baldlock 2008) after removal of carbonates; pH was measured in distilled water in the 1:2 (weight/volume) ratio (Hendershot et al. 2008).

Total contents of 13 elements (Ca, Cd, Co, Cu, Fe, K, Mg, Mn, Na, Ni, P, Pb, and Zn) in the copper tailings were determined by ICP-OES after microwave digestion in concentrated nitric acid with hydrogen peroxide oxidation (1.0 g of substrate treated with 10 mL of concentrated HNO_3_ and 2 mL H_2_O_2_, Merck Darmstadt). Appropriate Sigma-Aldrich ICP standards were used for working standards after matching a matrix.

Available forms of some elements (Ca, Co, Cu, Fe, K, Mg, Mn, P, and Zn) in air-dried and sieved (Ø 2-mm sieve) samples of the tailings and the soil were determined after extraction in 0.2 mol L^−1^ CH_3_COOH + 0.25 mol L^−1^ NH_4_Cl, 0.005 mol L^−1^ + C_8_H_8_O_7_ (citric acid) and 0.05 mol L^−1^ HCl pH = 1.3 by Yanai et al.’s (2000) method. The elements Mg, Fe, Mn, Zn, Cu, and Co were analyzed by atomic absorption spectrometry with a Varian SpectrAA 220 FS apparatus using an air-acetylene oxidizing flame, after optimization at specific wavelengths. Potassium and calcium were determined by atomic emission spectrometry with use of the air-acetylene flame for K detection, and the oxidizing nitrous oxide-acetylene flame for Ca. The caesium-lanthanum buffer (by Schinkel, Merck Darmstadt) was added as a releasing agent for K analysis. Colorimetric determination of phosphorus was done by (molybdenum blue) Murphy and Riley’s (1962) method with a Thermo Scientific Evolution 600 UV-Vis spectrophotometer at 715-nm wavelength.

Plant samples were dried and ground with a stainless steel cutter mill equipped with a 2-mm sieve. Next, 2.5 g of air-dried material was weighted into quartz crucibles and ashed in an oven, with a program ensuring slow increase of temperature to 500 °C, maintaining this temperature over 6 h, and then slowly cooling down. The ashed samples were dissolved in 6 mol dm^−3^ HCl (Sillanpää 1982), evaporated, and then transferred with 0.2 mol dm^−3^ HCl into a 50-mL volumetric flask. Element contents in plants were determined in conditions adequate for each element, as described above.

### Data analysis

Significance of differences between study sites in terms of element contents in substrates as well as plant species was evaluated with use of Student’s *t* test after verification of a normal distribution by Shapiro-Wilk’s *W* test and data Box-Cox transformation. The calculations were carried out with Statistica software version 10 (StatSoft 2011).

To reduce the amount of variables and for data exploration purposes, principal component analysis (PCA) was performed to analyze the variance in element contents in plants and redundancy analysis (RDA) was used to relate variation in the chemical composition of plants to the element contents in examined substrates. These linear methods of ordination were chosen after preliminary application of detrended correspondence analysis (DCA) of the plant data, which yielded axes of short gradients, i.e., below 0.4 SD (Lepš and Šmilauer 2003). In the PCA, a set of 13 elements and 37 samples of plant species from the copper tailings and the reference soil was analyzed. Student’s *t* test was used to assess the significance of differences between groups of the plant samples distinguished in the PCA ordination. The RDA was based on a 9 × 37 element-by-sample matrix, and the Monte Carlo permutation test (499 permutations under a reduced model) was performed to evaluate the statistical significance of constrained axes as well as of each descriptive variable in the model of regression of forward selection. Computations and ordination plots in PCA and RDA were made using CANOCO 4.55 for Windows software (ter Braak and Šmilauer 2002).

## Results

### Substrate properties

The granulometric composition of the copper tailings (Table 1) was characterized by the majority of a fraction with grains of a diameter lower than 0.05 mm. This fact indicates the unsuitable air-water conditions with the oxygen deficiency that occurred in wet seasons, which is particularly unfavorable for the plant root growth. Additionally, other authors (Spiak et al. 2012) reported that the tailings had an adverse water capacity because of too low content of the productive and easily accessible water (related to the matrix potential from 2.2 to 3.7 pF), which especially occurred in the top layer. Under such circumstances, the seedling mortality can be very high, which restricts plant settlement in a colonization process of the bare pond surface.

**Table 1:** Granulometric composition of the copper mine post-flotation tailings

	Granulometric fraction			
Grain size (mm)	> 2.0	0.05–2.0	0.002–0.05	< 0.002
Share (%)	0	1–13	63–85	5–35

The copper tailings had low content of organic carbon (< 0.3%) which mainly derived from black shale and lignite that are present in copper ore deposits, and from flotation agents. Due to the high content of carbonate minerals, they are characterized by an alkaline reaction with the pH_H2O_ value which ranged between 8.0 and 8.5. The pH value of the reference soil was in a range 5.8–6.2.

The total element contents found in the copper tailings are presented in Table 2. The mean phosphorus and potassium contents in the tailings were higher than the averages recorded for relatively poor Polish soils as well as European mean values, which results from the ore beneficiation in a flotation process. The total contents of Ca (16.3%) and Mg (2.4%) were very high in comparison to mean soil levels given by Lis et al. (2012) (0.47 and 0.10%, respectively) and Salminen et al. (2005) (2.53 and 1.04%, respectively). These occurrences are due to the fact that the tailings developed as assorted material from dolomite rocks. Among microelements, the total Cu content was extremely high (1800 mg kg^−1^) which is almost 13 times higher than the maximum allowable content of this element in European agricultural soils, as reported by Kabata-Pendias and Pendias (2001) (Cu < 140 mg kg^−1^) following EU legislation (CD 1986). This is despite the fact that the concentration of Cu decreases from about 2% in the mined ore to 0.18% in the deposited tailings. The total content of Co and Mn was very high too (20 and 1900 mg kg^−1^, respectively), and it exceeded the mean values of these elements for Polish as well as European soils, according to Lis et al. (2012) (3 and 267 mg kg^−1^, respectively) and Salminen et al. (2005) (10.4 and 627 mg kg^−1^, respectively). The levels of Co and Mn were also in accordance with the lower values of the allowable content of these elements in European agricultural soils, as given by Kabata-Pendias and Pendias (2001) (20 and 1500 mg kg^−1^, respectively); thus, the copper tailings can be considered as Co- and Mn-contaminated. The total Fe and Ni contents were a few times as high as Polish soil average values (6700 and 6.0 mg kg^−1^, respectively) (Lis et al. 2012), but they were not enriched in relation to European means. The total Pb level was comparable to the means determined for Polish and European soils. The obtained values of total Cd and Zn were lower than the means reported by Lis et al. (2012) (0.8 and 88 mg kg^−1^, respectively). The total Na content was much lower compared to the European mean.

**Table 2:** Total element content (mg kg^−1^) in the copper mine post-flotation tailings

	Ca	Cd	Co	Cu	Fe	K	Mg	Mn	Na	Ni	P	Pb	Zn
Mean	163,000	0.4	20.0	1800	18,000	27,000	24,000	1900	800	22.6	800	36.8	65.9
SD	5000	0.1	1.0	90	800	1000	1000	90	30	2.1	50	4.9	8.7

The chemical composition of the copper tailings and the reference soil differed significantly (*t* test, *P* < 0.05) in terms of content of the available forms of all analyzed elements (Table 3). The tailings were extremely rich in available Cu which was over 40 times as high as that of the soil. Levels of available Ca and Mg were very high too, and the calcium content was above seven times and magnesium content three times as high as that of the soil. Similarly, the content of available K, Fe, Co, and Mn was also considerably higher in the tailings. On the other hand, the copper tailings have several times lower content of the available P and a lower level of the available Zn, compared to the reference soil.

**Table 3:** Concentration of the available forms^a^ of elements (mg kg^−1^) in the copper mine post-flotation tailings and the reference soil

	Cu tailings		Reference soil		
	Mean ± SD	Median	Mean ± SD	Median	*P*
Ca	37,605 ± 1608	37,475	5129 ± 1620	4713	< 0.0001
Co	1.6 ± 0.2	2.0	0.8 ± 0.6	1.0	< 0.0002
Cu	745 ± 265	686	16 ± 13	10	< 0.0001
Fe	151 ± 11	155	94 ± 47	72	< 0.003
K	237 ± 55	239	133 ± 80	105	< 0.002
Mg	377 ± 57	355	112 ± 24	108	< 0.0001
Mn	318 ± 13	320	48 ± 8	48	< 0.0001
P	31 ± 11	30	712 ± 362	641	< 0.0001
Zn	2.2 ± 0.9	2.0	17 ± 11	15	< 0.0001

The results are presented as mean with standard deviation (SD) and median; *t* test probability level (*P*) for comparison of means of both substrates, significant differences in bold text

^a^Determined according to the Yanai et al. 2000

### Element content in plants

A comparison of chemical composition of plant shoots collected from the copper tailings and the reference soil revealed the occurrence of many considerable differences (Table 4). The plants from copper tailings, except for *T. farfara*, had higher Ca content. The highest mean calcium value was found in *C. arvense* (29 g kg^−1^), and this was about seven times as high as that from the soil. As opposed to the other species, *T. farfara* accumulated calcium at a very high level (21 g kg^−1^), which was the same in both study sites. The shoot Mg content was considerably higher in the plants from tailings, and *P. aviculare*, *C. arvense*, and *T. farfara* accumulated magnesium above the requirement for optimal plant growth, according to Marschner (2012) (1.5–3.5 g kg^−1^). Among the species growing in tailings, grasses, i.e., *A. stolonifera* and *C. epigejos*, were characterized by the lowest Ca and Mg levels. The plants from copper tailings, relative to those from the soil, accumulated phosphorus at the same level as in the case of dicotyledonous, or had a significantly lower P content (*t* test, *P* < 0.05) than in both grass species with the lowest result noted in *C. epigejos*. The K accumulation pattern differed widely among the studied sites and the species. The plants from tailings had much higher Na content with the highest result (78.7 mg kg^−1^) found in *P. aviculare* which accumulated 17 times as much sodium as that from the soil.

**Table 4:** Element content (mg kg^−1^) in plant shoots from the copper mine post-flotation tailings and the reference soil

	Study site		*Agrostis stolonifera* L.	*Calamagrostis epigejos* (L.) Roth	*Cerastium arvense* L.	*Polygonum aviculare* L.	*Tussilago farfara* L.
Ca	Cu tailings	Mean ± SD Median	6840 ± 1457 7100	7600 ± 2700 6150	29000 ± 2800 28900	19100 ± 1700 19400	21000 ± 1900 20100
	Reference soil	Mean ± SD Median	4030 ± 651 4000	4100 ± 960 4500	3800 ± 1200 3888	6500 ± 1300 7100	21500 ± 1900 21600
		*P*	*< 0.02*	> 0.05	*< 0.0001*	*< 0.0001*	> 0.05
Cd	Cu tailings	Mean ± SD Median	0.36 ± 0.10 0.41	0.80 ± 0.10 0.81	0.72 ± 0.05 0.70	0.61 ± 0.13 0.60	0.28 ± 0.03 0.26
	Reference soil	Mean ± SD Median	0.34 ± 0.06 0.32	0.30 ± 0.03 0.30	0.52 ± 0.66 0.24	0.33 ± 0.03 0.33	0.33 ± 0.03 0.34
		*P*	> 0.05	< *0.001*	> 0.05	< *0.02*	> 0.05
Co	Cu tailings	Mean ± SD Median	1.50 ± 0.30 1.62	1.19 ± 0.30 1.13	3.90 ± 0.50 3.98	2.70 ± 0.20 2.71	3.10 ± 0.20 3.06
	Reference soil	Mean ± SD Median	1.30 ± 0.20 1.40	0.97 ± 0.10 0.97	0.93 ± 0.10 0.92	1.40 ± 0.10 1.41	2.70 ± 0.10 2.67
		*P*	> 0.05	> 0.05	*< 0.0001*	*< 0.0001*	> 0.05
Cu	Cu tailings	Mean ± SD Median	10.40 ± 1.40 10.70	6.00 ± 1.90 6.08	47.00 ± 8.50 43.80	37.80 ± 5.50 39.00	16.70 ± 4.80 11.80
	Reference soil	Mean ± SD Median	4.60 ± 1.30 4.80	2.70 ± 0.80 2.86	5.70 ± 1.10 5.83	3.80 ± 0.10 3.78	5.40 ± 1.70 4.60
		*P*	*< 0.001*	*< 0.04*	*< 0.0001*	*< 0.0001*	*< 0.02*
Fe	Cu tailings	Mean ± SD Median	38.30 ± 5.30 40.00	30.67 ± 5.20 31.00	95.10 ± 25.40 82.00	68.10 ± 17.20 69.00	54.40 ± 13.50 47.00
	Reference soil	Mean ± SD Median	103.10 ± 27.80 114.00	57.54 ± 8.80 53.30	125.90 ± 54.30 114.00	370.00 ± 94.00 407.00	92.90 ± 17.30 102.00
		*P*	*< 0.002*	*< 0.004*	> 0.05	*< 0.001*	*< 0.04*
K	Cu tailings	Mean ± SD Median	16000 ± 1420 15600	12000 ± 1200 12000	36800 ± 9900 33100	18900 ± 2800 18250	39600 ± 2400 38900
	Reference soil	Mean ± SD Median	25000 ± 6450 23400	15600 ± 2900 15400	25100 ± 7000 23375	7800 ± 1200 9820	28100 ± 1000 27900
		*P*	*< 0.02*	> 0.05	*< 0.02*	*< 0.001*	*< 0.002*
Mg	Cu tailings	Mean ± SD Median	1560 ± 40 1600	1350 ± 170 1400	7300 ± 1400 6800	7800 ± 1500 7550	5000 ± 1700 5300
	Reference soil	Mean ± SD Median	1300 ± 30 1300	830 ± 150 800	2400 ± 700 2510	930 ± 60 900	2600 ± 900 2500
		*P*	> 0.05	*< 0.009*	*< 0.0001*	*< 0.001*	> 0.05
Mn	Cu tailings	Mean ± SD Median	47.40 ± 10.30 52.00	118.60 ± 34.90 136.00	126.60 ± 30.00 129.00	128.80 ± 24.60 117.00	22.20 ± 6.30 21.00
	Reference soil	Mean ± SD Median	55.70 ± 4.90 57.00	35.70 ± 13.70 35.00	84.30 ± 55.30 55.00	45.00 ± 11.60 39.00	41.60 ± 3.20 42.00
		*P*	> 0.05	*< 0.01*	> 0.05	*< 0.003*	> 0.05
Na	Cu tailings	Mean ± SD Median	23.40 ± 2.60 22.80	16.20 ± 2.50 15.70	58.90 ± 18.40 55.00	78.70 ± 18.10 82.00	42.90 ± 12.00 39.00
	Reference soil	Mean ± SD Median	4.90 ± 1.20 5.30	14.80 ± 1.70 15.40	21.30 ± 9.30 22.00	4.60 ± 0.10 4.65	38.90 ± 6.60 39.00
		*P*	*< 0.00003*	> 0.05	*< 0.0001*	*< 0.01*	> 0.05
Ni	Cu tailings	Mean ± SD Median	4.97 ± 1.63 4.28	5.40 ± 0.80 5.30	5.05 ± 0.58 4.90	4.96 ± 1.07 4.77	3.02 ± 0.57 3.40
	Reference soil	Mean ± SD Median	3.41 ± 0.35 3.30	2.20 ± 0.20 2.20	0.63 ± 0.35 0.60	3.05 ± 0.09 3.09	3.05 ± 0.09 4.10
		*P*	> 0.05	< *0.0009*	< *0.0000005*	< *0.03*	> 0.05
P	Cu tailings	Mean ± SD Median	1160 ± 30 1100	950 ± 200 850	2200 ± 400 2100	2200 ± 200 2150	1830 ± 150 1800
	Reference soil	Mean ± SD Median	3430 ± 110 3300	2500 ± 300 2500	2700 ± 1100 2885	2200 ± 200 2300	1830 ± 290 2000
		*P*	*< 0.004*	*< 0.003*	> 0.05	> 0.05	> 0.05
Pb	Cu tailings	Mean ± SD Median	5.71 ± 1.54 6.40	11.60 ± 1.10 11.40	8.79 ± 2.40 7.72	8.12 ± 1.89 7.60	3.65 ± 0.39 3.50
	Reference soil	Mean ± SD Median	4.18 ± 0.62 3.85	3.60 ± 0.30 3.55	3.46 ± 0.22 3.44	5.02 ± 0.08 5.00	6.35 ± 0.43 6.40
		*P*	> 0.05	< *0.0001*	< *0.001*	< *0.04*	< *0.002*
Zn	Cu tailings	Mean ± SD Median	28.80 ± 8.30 28.80	17.10 ± 4.60 15.10	35.80 ± 8.70 37.00	39.40 ± 7.70 36.00	20.50 ± 3.5020.00
	Reference soil	Mean ± SD Median	25.70 ± 11.20 21.70	23.70 ± 9.80 23.10	61.40 ± 11.0 59.00	34.30 ± 2.20 33.00	31.80 ± 4.90 29.00
		*P*	> 0.05	> 0.05	> 0.05	> 0.05	*< 0.03*

The results are presented as values of mean ± standard deviation (SD) and Median, *t* test probability level (*P*) for comparison of means of both study sites, significant differences  are italicized

The shoot Cu content was significantly higher (*t* test, *P* < 0.05) in all plants from tailings compared to those growing in the reference soil. The highest amounts were found in *C. arvense* and *P. aviculare* (47.0 and 37.8 mg kg^−1^, respectively). These values were nearly eight and ten times as high as those of the soil environment, and they were within the range of critical toxicity Cu level for plants, according to Kabata-Pendias and Pendias (2001) (20–100 mg kg^−1^) as well as the European Union legislation (EU 2008). The experimental critical toxic values for cultivating plants were estimated to be 8–40 mg Cu kg^−1^ (Davis and Beckett 1978; Macnicol and Beckett 1985), but we cannot state unambiguously if the toxic effect occurs in the species mentioned above because relevant data are rather scarce.

All species from tailings accumulated cadmium and cobalt above normal values for plants (0.2 and 1.0 mg kg^−1^, respectively) but below the toxic levels, as given by Kabata-Pendias and Pendias (2001). The highest Cd amounts were found in shoots of *C. epigejos*, *C. arvense*, and *P. aviculare* (0.8–0.6 mg kg^−1^, respectively). The species from tailings were distinctly enriched in Ni and Pb whose contents approached the maximum of the normal ranges (5 and 10 mg kg^−1^, respectively; Kabata-Pendias and Pendias 2001). The Mn content was within the normal values for plants in all studied species. As opposed to the others, *T. farfara* did not accumulate higher amounts of Cd, Ni, Mn, and Pb growing in the tailings environment.

The shoot Fe content was much lower in all species growing in tailings compared to those from the soil, and statistically significant differences (*t* test, *P* < 0.05) were found in most cases. The iron levels for the examined grasses were below the range of critical deficiency concentration (50–150 mg Fe kg^−1^, according to Marschner (2012)), and the lowest result was found in *C. epigejos*. The shoot Zn accumulation showed low variability among study sites and plant species. The lowest Zn level obtained in *C. epigejos* (17.1 mg kg^−1^) was within a range of the critical deficiency concentration given by Marschner (2012) (5–20 mg kg^−1^).

In PCA, the highest eigenvalues were achieved for the first two principal components (0.603 and 0.133, respectively) which explained 73.6% in total of the variability in shoot element contents. The first principal component was strongly determined by Cu, Mg, Pb, Na, Ca, and Co with the linear correlation coefficient *r* ranging from 0.96 (Cu) to 0.85 (Co), respectively; the influence of Ni was much weaker (*r* = 0.56). The second component mainly represented the gradient of Fe, P, and Zn (*r* = 0.91 to 0.62, respectively). Values of the variables which determine the first and second principal components are linearly uncorrelated.

**Fig. 2:**
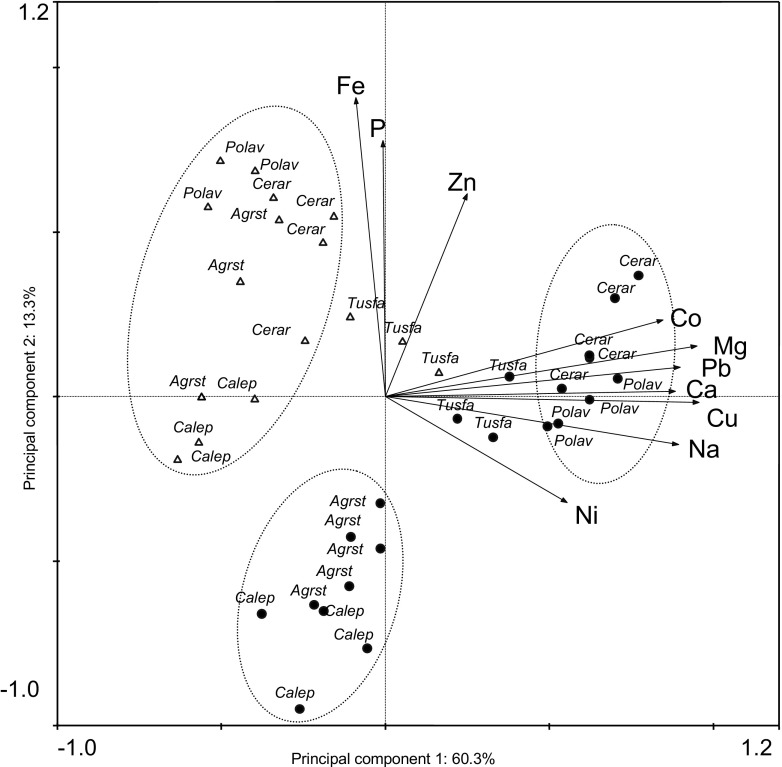
Principal component analysis (PCA) ordination plot based on the accumulation of P, K, Ca, Mg, Na, Cd, Co, Cu, Fe, Mn, Ni, Pb, and Zn in shoots of plants from the copper post-flotation tailings and the reference soil, with groups of species that differ in the element contents. The variables with no significant effect (<0.5 correlation  coefficient with axes) are not presented. *Agrst*—*Agrostis stolonifera*, *Calep—Calamagrostis epigejos*, *Cerar— Cerastium arvense*, *Polav—Polygonum aviculare*, *Tusfa—Tussilago farfara*; filled circle—copper tailings, open triangle—reference soil

The PCA ordination (Fig. 2) indicated a few groups of plant samples distinguished by the gradient of the first and second factors. The plant samples from the reference soil, except for *T. farfara*, formed one group where the first axis returned negative scores (the lowest tissue content of Cu, Mg, Pb, Na, Ca, Co, and Ni) and the second axis returned high positive or close to zero scores (the highest and near to medium Fe, P, and Zn contents). The plant samples from tailings were divided into two clearly separated groups of various species. The first one consisted of *C. arvense* and *P. aviculare* which had the highest positive scores of the first axis (the highest or high Cu, Mg, Pb, Na, Ca, Co, and Ni contents) and positive or close to zero scores of the second axis (high and medium levels of Fe, P, and Zn). The second one included *A. stolonifera* and *C. epigejos* which differed from the other samples from tailings by the lower scores of the first axis and had the lowest scores of the second axis. These two grasses accumulated significantly lower amounts of most elements considerable in this PCA model i.e., Cu, Mg, Na, Ca, Co, Fe, P, and Zn, in relation to *C. arvense* and *P. aviculare* growing in tailings (*t* test, *P* < 0.0001) as well as *T. farfara* but in this case without Zn (*t* test, *P* < 0.0001 to < 0.02, respectively). These species also differed significantly in levels of Fe, P, Cu, Ca, Ni Na, and Zn (*t* test, *P* < 0.00001 to < 0.04, respectively) compared to the group of samples from the reference soil. The samples of *T. farfara*, both from the soil and the copper tailings, were placed in a central part of the diagram and were not sharply separated from each other or from the other samples. *T. farfara* from soil differed significantly from the other species growing in the same environment by higher contents of Ca, Co, Pb, and Mg (*t* test, *P* < 0.0001–0.04), whereas *T. farfara* from tailings contained considerably lower amounts of Mn, Zn, Cu, Mg, and Pb than *C. arvense* and *P. aviculare* from the same study site (*t* test, *P* < 0.0001–0.031, respectively).

### Relation of element content in plants to the concentration in substrates

Redundancy analysis (RDA) demonstrated a significant relationship between element contents in plants and concentrations of the available forms of elements in substrates. In the resulting RDA model, nine tested explanatory variables, i.e., the concentration of available P, K, Ca, Mg, Fe, Cu, Co, Mn, and Zn in substrates, explained 43.5% of the total variability in the element contents in plant samples. The first canonical axis accounted for 33.5% of the variation and was highly statistically significant (Monte Carlo permutation test, *P* = 0.002), whereas the second axis was not significant in this model. The forward stepwise selection of variables revealed that each of the subset of the explanatory variables, i.e., the concentration of Mn, Ca, Zn, Mg, Cu, and P, was statistically significant (Monte Carlo permutation test, *P* = 0.002) when tested independently and explained the variation at the comparable level, Lambda 1 = 0.31 (Mn) to 0.27 (P). In addition, all these six descriptors were highly cross-correlated (all correlation coefficients > 0.83), but the values of Mn, Ca, Mg, and Cu were mutually negatively related to Zn and P. The explanatory effect of Co concentration in substrates was also significant but lower than others (Lambda 1 Co = 0.17, *P* = 0.002). The effect of K and Fe levels in substrates appeared to be insignificant.

**Fig. 3:**
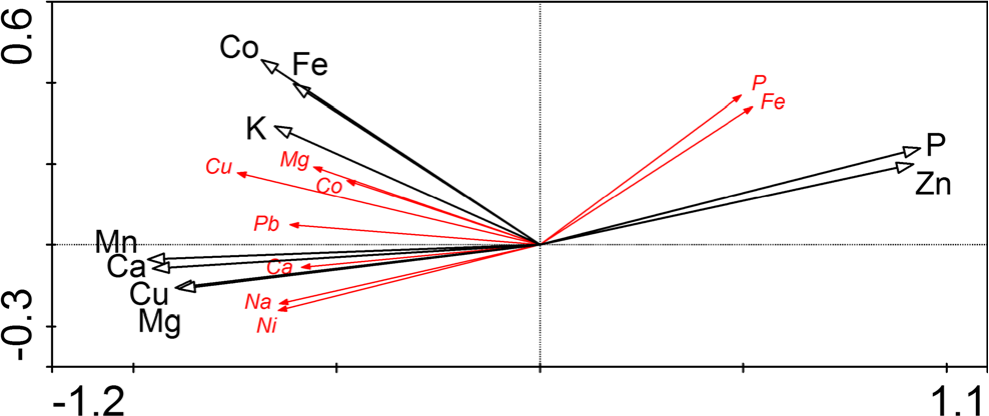
Redundancy analysis (RDA) ordination plot—the effect of Fe, Ca, Co, Cu, K, Mg, Mn, P, and Zn concentration in the copper tailings and the reference soil on the content of some elements in plants (according to PCA). The less significant variables (< 0.5 correlation coefficient with axes) are not presented. Black and red vectors indicate the explanatory and the response variables, respectively

The RDA diagram (Fig. 3) presents the ordination of the nutrient contents in plants along gradients of the element concentrations in substrates. The location and length of vectors in the ordination space indicate the relations and their strength. It was noticeable that the high levels of Mn, Ca, Mg, Cu, and Co that occurred in the copper tailings were mutually significantly correlated with the high amounts of Cu, Ni, Na, Pb, Ca, Mg, Co, and Mn in plants (the negative part of the first axis). On the other side, the higher levels of available P and Zn, characteristic of the reference soil, were mutually significantly related with the higher Fe and P contents in plants. Additionally, there was no significant correlation between the content of Fe and Zn in the studied substrates and plants, and all associations of Cd and K contents in plants with each other variable were insignificant.

## Discussion

The obtained results revealed that plant species growing in the tailings and the reference soil varied significantly in shoot element contents and that plant accumulation patterns depended on the site conditions as well as systematic position of the plant, and the latter occurred distinctly in the specific copper tailings environment. It was also found that the contents of the available Ca, Mg, P, Cu, Co, and Mn in the studied substrates were significantly and positively correlated with the content of these elements in the plant shoots while correlations for the available Fe and Zn were insignificant.

Under the same site conditions, the shoot calcium content differs considerably between plant species and higher phylogenetic units (Broadley et al. 2003), and monocotyledons, including the Poaceae family, have generally lower Ca and Mg shoot contents than dicotyledons (Thompson et al. 1997). This corresponds to our findings of the significantly lower levels of these elements in *A. stolonifera* and *C. epigejos* from the tailings environment that is highly enriched in these elements. The ability to accumulate Ca in the shoots is also determined by plant physiotype. Among the oxalate plants, members of the Caryophyllaceae and Polygonaceae family increase the shoot Ca content proportionally to increasing Ca supply (Kinzel and Lechner 1992). A similar phenomenon was also observed in *C. arvense* and *P. aviculare* in our study. Furthermore, *C. arvense* can detoxify the Mg surplus in sparingly soluble oxalate salt (Kinzel and Weber 1982; Popp 1983). It was also demonstrated that *T. farfara*, as a calcium accumulator, took up Ca in spite of its low concentration in nutrient solution and the uptake and growth of this species were stimulated by higher Ca ion supply (Kinzel and Lechner 1992). These results can explain the high Ca accumulation levels found in this species both in the reference soil and in the copper tailings.

Both *P. aviculare* and *C. arvense* growing in the copper tailings were characterized by the highest shoot Cu content as well as comparatively high levels of all studied trace elements, which implies their high metal accumulation capacity. Similarly, other authors also found high levels of Zn, Cd, Cu, and Pb in shoots of *P. aviculare* growing on Zn tailings (Carrillo-Gonzales and Gonzales-Chavez 2006) and in an urban environment (Polechońska et al. 2013). Additionally, high efficiency of the shoot Na accumulation confirms the salt tolerance of this species demonstrated in the other study (Arnaud and Vincent 1988). *C. arvense* and other members of Caryophyllaceae are important components of serpentine vegetation, and they exhibit adaptation features to colonize these specific substrata (Kasowska and Koszelnik-Leszek 2014) together with tolerance mechanisms of high metal levels (Kazakou et al. 2008). It was found that this species accumulated shoot Ni at relatively moderate levels (Lombini et al. 1998).

The studied grass species from the copper tailings, besides the lower shoot Ca and Mg levels, accumulated significantly lower amounts of other elements as Cu, Co, and Na than the examined species of dicotyledons. Generally, grasses are regarded as metal excluders, as defined by Baker (1981). *A. stolonifera* can evolve tolerant populations that have a greater capacity to accumulate copper in the roots and restrict transport of this element to the shoots, probably due to the existence of an efficient copper-complexing mechanism (Wu et al. 1975). This species may also evolve magnesium and salt tolerance, and the last with Na exclusion mechanism from roots and shoots (Wu 1981). Likewise, Fitzgerald et al. (2003) obtained lower Cu content in shoots of *A. stolonifera* compared to the roots as well as the shoots of dicotyledons. Similarly, *C. epigejos* growing on ash deposits accumulated much less Cu in the shoots than in the roots (Mitrović et al. 2008), and the shoot Cu level was comparable to that obtained in our study. Lehmann and Rebele (2004) stated that the copper tolerance of the *C. epigejos* population from a copper smelter was at a comparable level to that of copper-tolerant *A. stolonifera*.

Accumulation of cobalt was below the critical toxicity level in all studied species despite of its high content in the copper tailings, which implies reduced bioavailability of this element in this substrate. The solubility and toxicity of Co to plants decrease with an increase in the exchangeable Ca content in the soil solution (Li et al. 2009). This probably occurs in the tailings, because of their very high total and available Ca contents. The uptake and distribution of Co in plants is species-dependent, and this element is accumulated mainly in the roots (Palit et al. 1994; Page and Feller 2005). However, some studies reported that higher amounts of cobalt were retained in the root systems of monocotyledons while its transport from the roots to the shoots in dicotyledons was highly effective (Bakkaus et al. 2005; Collins et al. 2010).

Low amounts of phosphorus were detected in shoots of the species from copper tailings despite of its high total content in this substrate. The low bioavailability of this element can be attributed to high pH, and the abundance of calcium-magnesium carbonates occurred in the tailings. The excess of carbonates intensifies P sorption on their surface, and the high content of Ca and Mg increases phosphorus precipitation in the soil solution as Ca-Mg phosphates (Lindsay 1979; Sharpley et al. 1989) which are poorly soluble at high pH [(Ca_3_(PO_4_)_2_ Ksp = 1.4 × 10^29^, Mg_3_(PO_4_)_2_ Ksp = 6.3 × 10^26^] (Corbridge 2013). It is known that plant species and their genotypes can develop a wide range of adaptive mechanisms involved in phosphorus acquisition (Ramaekers et al. 2010). However, especially at low P availability, enhanced P uptake could be achieved by releasing protons, carboxylates, and other exudates into the rhizosphere (Lambers et al. 2015). For instance, in alkaline and calcareous soils, oxalic acid compared to other organic anions was considered to be the most effective in mobilizing unavailable phosphorus (Ström et al. 2005; Wang and Chen 2015). It can be suggested that the very low shoot P content obtained in *A. stolonifera* and *C. epigejos* in the copper tailings is indicative of the little effective P-mobilizing strategy used by these grasses under calcareous conditions. Additionally, diminishing in P uptake effectiveness in the tailings habitat could also be caused by lack of arbuscular mycorrhiza (AM), because this symbiosis commonly occurs in both these species and was found in their roots in the reference soil (unpublished data). In the copper tailings, typically mycorrhizal species *A. stolonifera*, *C. epigejos*, and *T. farfara* realized P uptake only by their root mechanisms, which resulted in P deficiency in the case of the grasses.

The alkaline soil environment strongly modifies the Fe uptake (Schenkeveld et al. 2014b), and the increase in pH by one unit diminishes Fe solubility 1000-fold, which can induce Fe deficiency aggravated by the presence of free carbonates (Coulombe et al. 1984; Inskeep and Bloom 1984). As a result, much lower shoot Fe contents were found in all species growing in the copper tailings compared to the reference soil, with the critical deficiency concentration occurring in both grasses. In neutral and calcareous soils with Fe deficiency, grasses excrete phytosiderophores (PS) for iron acquisition that in turn form soluble Fe complexes easily taken up (Römheld and Marschner 1986; Reichman and Parker 2005). PS can also mobilize other trace elements, particularly Cu, Zn, Ni, Co, Cd, and Mn (Romheld 1991; Schenkeveld et al. 2014a, b). This can increase their bioavailability but also may result in competition between Fe(III) and other metals for binding to PS complexes. Especially, the mobilization of Cu can diminish solubilized Fe(III) content (Reichman and Parker 2005), which might aggravate the iron deficiency that occurred in both grass species in tailings conditions. Inputs of PS into the rhizosphere strongly depend on the Fe status of the plant, the species, and the genotype (Bashir et al. 2006; Pereira et al. 2014); the diurnal PS release cycle; and other factors (Reichman and Parker 2005). Thus, the mechanisms involved in strategy II of Fe uptake and interactions with other elements could result in the lowest iron content found in shoots of *A. stolonifera* and *C. epigejos* as well as in differences in levels of accumulation of other metals that were present in these both grass species in tailings conditions.

Reaction of the soil solution also affects manganese availability but to a lesser extent than in the case of iron. According to Sanders (1983), decreasing the acidity from pH 5.2 to 7.3 reduces Mn solubility over 200-fold. Then also the high content of both total and available Ca and Mg forms acts antagonistically to Mn uptake (Maas et al. 1969) and the adsorption of Mn on carbonate surfaces diminishes the amount of soluble manganese (McBride 1979). However, besides decreased manganese bioavailability, the Mn content in the plants from copper tailings was higher in the same species compared to the reference soil, but this differentiation of Mn levels was without significant relation to the groups distinguished by PCA, and high Mn levels were found in *C. arvense*, *P. aviculare*, and *C. epigejos*. According to Lambers et al. (2015), plants that release carboxylates in their phosphorus-acquisition strategy tend to have high leaf manganese concentrations because of possibility of Mn mobilization even under its low availability. This association between considerable leaf Mn levels and carboxylate release in P-impoverished habitats was suggested for plants without arbuscular mycorrhiza even at lack of a significant correlation between shoot Mn and P concentrations (Oliveira et al. 2015). Similarly, the absence of this correlation could also be observed in RDA in our study. In this connection, the high shoot Mn level found together with the lowest *P* value in *C. epigejos* from the copper tailings implies that this species has particular difficulties in P uptake in the tailings environment.

The copper tailings had low zinc content. Additionally, the uptake of this nutrient is also limited at high pH and carbonate content and clay minerals can strongly absorb this element (Alloway 2009; Lambers et al. 2008). The lowest zinc level found in *C. epigejos* from tailings was comparable to the result obtained on alkaline ash deposits (Mitrović et al. 2008). It was also noted that this species growing on Zn-Pb wastes had the lowest shoot Zn content compared to other species (Wójcik et al. 2014). Exudation of phytosiderophores in grasses or carboxylates can mobilize Zn in alkaline conditions (Lambers et al. 2015). However, Marschner (2012) also lists other solutes excreted by dicotyledonous at Zn deficiency. In our study, the shoot P, Fe, and Zn contents were significantly correlated with a single principal component which distinguished *A. stolonifera* and *C. epigejos* growing in tailings from other plants by the lowest content of these elements. This implies a similar mode of acquisition mechanisms of these nutrients in these species which proved very little effective in the copper tailings conditions.

The investigation contributes towards a better understanding of the mechanisms used by plants to respond to specific properties of tailings from the copper mining industry in an effort to develop an efficient strategy for their remediation. The most suitable plant species for this process proved to be *A. stolonifera* due to its demonstrated low shoot heavy metal content, which is important in the context of entering these elements into the food chain. In contrast to *C. epigejos*, creeping bent grass also acquired limiting nutrients P, Fe, and Zn more efficiently, which can result in a competitive advantage of this species in a plant community on the tailings. Considering also our earlier results (Spiak et al. 2012), in the phytostabilization process we propose to apply any kind of amendment (e.g., sand) to improve the tailings structure and the air-water relations at least in the surface layer, and to sow legumes from *Trifolium* and *Medicago* genera using seeds inoculated with *Rhizobium* bacteria. As hosts for rhizobia and arbuscular mycorrhizal fungi, these plants can fix nitrogen and uptake phosphorus more effectively, which enhances both their growth and that of neighboring plants. It seems that legumes could effectively colonize the tailings because high Ca and Co contents enhance their nodule formation (Smit et al. 1989; Arrigioni et al. 2013; Kliewer and Evans 1963). Before establishing AM, phosphorus fertilization should be used to stimulate plant growth and symbiotic associations. Following the vegetation development, processes of acidification usually intensify. This can trigger releasing higher amounts of copper and other potentially toxic metals into the tailings environment. Thus, monitoring of the site properties, the plant responses, and the presence and activity of microorganisms will be required for the evaluation of effectiveness of the remediation.

## Conclusions

The tailings from the copper mining industry had a lot of unfavorable features for vegetation development, especially the adverse air-water conditions, the low availability of nutrients such as P, Fe, and Zn, and a very high content of Cu, Co, and Mn. The chemical composition of plants colonizing the tailings reflected the concentration of the available forms of Ca, Mg, P, Cu, Co, and Mn, but the species differed distinctly in their response to the tailings properties. *Polygonum aviculare* and *Cerastium arvense* had high shoot accumulation capacity of all studied elements with the highest Cu contents lying within the range of critical toxicity for plants, whereas the grass species had lower levels of majority of elements, especially Cu, P, Fe, and Zn. *Agrostis stolonifera* proved to be the most suitable species for phytostabilization of the tailings with regard to its low shoot heavy metal content and, as opposed to *Calamagrostis epigejos*, more efficient acquisition of the limiting nutrients. The remediation process of the tailings, in view of potentially high costs, should at least involve the application of amendments improving their texture, phosphorus fertilization, and the introduction of native leguminous species.
