# Correction to: Heavy metal and nutrient uptake in plants colonizing post-flotation copper tailings

**DOI:** 10.1007/s11356-017-0766-8

**Published:** 2017-11-28

**Authors:** Dorota Kasowska, Krzysztof Gediga, Zofia Spiak

**Affiliations:** 1 Department of Botany and Plant Ecology, Wrocław University of Environmental and Life Sciences, Grunwaldzki Square 24A, 50-363 Wrocław, Poland; 2 Department of Plant Nutrition, Wrocław University of Environmental and Life Sciences, Grunwaldzka Street 53, 50-357 Wrocław, Poland

**Correction to: Environ Sci Pollut Res**

10.1007/s11356-017-0451-y

The correct presentation of Table 4 is shown in this paper.

**Table 4:** Element content (mg kg^-1^) in plant shoots from the copper mine post-flotation tailings and a reference soil. The results are presented as values of ± means standard deviation (SD) and median, t test probability level (P) for comparison of means of both study sites, significant differences in bold text

	Study site		*Agrostis stolonifera* L.	*Calamagrostis epigejos* (L.) Roth	*Cerastium arvense* L.	*Polygonum aviculare* L.	*Tussilago farfara* L.
Ca	Cu-tailings	Mean ± SD Median	6840±150 7100	7600±2700 6150	29000±2800 28900	19100±1700 19400	21000±1900 20100
	reference soil	Mean ± SD Median	4030±600 4000	4100±960 4500	3800±1200 3888	6500±1300 7100	21500±1900 21600
		*P*	*<0.02*	>0.05	*<0.0001*	*<0.0001*	>0.05
Cd	Cu-tailings	Mean ± SD Median	0.36±0.10 0.41	0.80±0.10 0.81	0.72±0.05 0.70	0.61±0.13 0.60	0.28±0.03 0.26
	reference soil	Mean ± SD Median	0.34±0.06 0.32	0.30±0.03 0.30	0.52±0.66 0.24	0.33±0.03 0.33	0.33±0.03 0.34
		*P*	>0.05	*<0.001*	>0.05	*<0.02*	>0.05
Co	Cu-tailings	Mean ± SD Median	1.50±0.30 1.62	1.19±0.30 1.13	3.90±0.50 3.98	2.70±0.20 2.71	3.10±0.20 3.06
	reference soil	Mean ± SD Median	1.30±0.20 1.40	0.97±0.10 0.97	0.93±0.10 0.92	1.40±0.10 1.41	2.70±0.10 2.67
		*P*	>0.05	>0.05	*<0.0001*	*<0.0001*	>0.05
Cu	Cu-tailings	Mean ± SD Median	10.40±1.40 10.70	6.00±1.90 6.08	47.00±8.50 43.80	37.80±5.50 39.00	16.70±4.80 11.80
	reference soil	Mean ± SD Median	4.60±1.30 4.80	2.70±0.80 2.86	5.70±1.10 5.83	3.80±0.10 3.78	5.40±1.70 4.60
		*P*	*<0.001*	*<0.04*	*<0.0001*	*<0.0001*	*<0.02*
Fe	Cu-tailings	Mean ± SD Median	38.30±5.30 40.00	30.67±5.20 31.00	95.10±25.40 82.00	68.10±17.20 69.00	54.40±13.50 47.00
	reference soil	Mean ± SD Median	103.10±27.80 114.00	57.54±8.80 53.30	125.90±54.30 114.00	370.00±94.00 407.00	92.90±17.30 102.00
		*P*	*<0.002*	*<0.004*	>0.05	*<0.001*	*<0.04*
K	Cu-tailings	Mean ± SD Median	16000±140 15600	12000±1200 12000	36800±9900 33100	18900±2800 18250	39600±2400 38900
	reference soil	Mean ± SD Median	25000±640 23400	15600±2900 15400	25100±7000 23375	7800±1200 98200	28100±1000 27900
		*P*	*<0.02*	>0.05	*<0.02*	*<0.001*	*<0.002*
Mg	Cu-tailings	Mean ± SD Median	1560±40 1600	1350±170 1400	7300±1400 6800	7800±1500 7550	5000±1700 5300
	reference soil	Mean ± SD Median	1300±30 1300	830±150 800	2400±700 2510	930±60 900	2600±900 2500
		*P*	>0.05	*<0.009*	*<0.0001*	*<0.001*	>0.05
Mn	Cu-tailings	Mean ± SD Median	47.40±10.30 52.00	118.60±34.90 136.00	126.60±30.00 129.00	128.80±24.60 117.00	22.20±6.30 21.00
	reference soil	Mean ± SD Median	55.70±4.90 57.00	35.70±13.70 35.00	84.30±55.30 55.00	45.00±11.60 39.00	41.60±3.20 42.00
		*P*	>0.05	*<0.01*	>0.05	*<0.003*	>0.05
Na	Cu-tailings	Mean ± SD Median	23.40±2.60 22.80	16.20±2.50 15.70	58.90±18.40 55.00	78.70±18.10 82.00	42.90±12.00 39.00
	reference soil	Mean ± SD Median	4.90±1.20 5.30	14.80±1.70 15.40	21.30±9.30 22.00	4.60±0.10 4.65	38.90±6.60 39.00
		*P*	*<0.00003*	>0.05	*<0.0001*	*<0.01*	>0.05
Ni	Cu-tailings	Mean ± SD Median	4.97±1.63 4.28	5.40±0.80 5.30	5.05±0.58 4.90	4.96±1.07 4.77	3.02±0.57 3.40
	reference soil	Mean ± SD Median	3.41±0.35 3.30	2.20±0.20 2.20	0.63±0.35 0.60	3.05±0.09 3.09	3.05±0.09 4.10
		*P*	>0.05	*<0.0009*	*<0.0000005*	*<0.03*	>0.05
P	Cu-tailings	Mean ± SD Median	1160±30 1100	950±200 850	2200±400 2100	2200±200 2150	1830±150 1800
	reference soil	Mean ± SD Median	3430±110 3300	2500±300 2500	2700±1100 2885	2200±200 2300	1830±290 2000
		*P*	*<0.004*	*<0.003*	>0.05	>0.05	>0.05
Pb	Cu-tailings	Mean ± SD Median	5.71±1.54 6.40	11.60±1.10 11.40	8.79±2.40 7.72	8.12±1.89 7.60	3.65±0.39 3.50
	reference soil	Mean ± SD Median	4.18±0.62 3.85	3.60±0.30 3.55	3.46±0.22 3.44	5.02±0.08 5.00	6.35±0.43 6.40
		*P*	>0.05	*<0.0001*	*<0.001*	*<0.04*	*<0.002*
Zn	Cu-tailings	Mean ± SD Median	28.80±8.30 28.80	17.10±4.60 15.10	35.80±8.70 37.00	39.40±7.70 36.00	20.50±3.50 20.00
	reference soil	Mean ± SD Median	25.70±11.20 21.70	23.70±9.80 23.10	61.40±11.0 59.00	34.30±2.20 33.00	31.80±4.90 29.00
		*P*	>0.05	>0.05	>0.05	>0.05	*<0.03*

The original article has been corrected.

